# 30-Day Mortality in Acute Pulmonary Embolism: Prognostic Value of Clinical Scores and Anamnestic Features

**DOI:** 10.1371/journal.pone.0148728

**Published:** 2016-02-11

**Authors:** Andreas Gunter Bach, Bettina-Maria Taute, Nansalmaa Baasai, Andreas Wienke, Hans Jonas Meyer, Dominik Schramm, Alexey Surov

**Affiliations:** 1 Department of Radiology, Martin-Luther-University Halle-Wittenberg, Ernst-Grube-Str. 40, 06120 Halle, Germany; 2 Department of Internal Medicine, Martin-Luther-University Halle-Wittenberg, Ernst-Grube-Str. 40, 06120 Halle, Germany; 3 Institute of Medical Epidemiology, Biostatistics and Informatics; Martin-Luther-University Halle-Wittenberg, Magdeburger-Str. 8, 06112 Halle, Germany; University of Colorado Denver, UNITED STATES

## Abstract

**Purpose:**

Identification of high-risk patients with pulmonary embolism is vital. The aim of the present study was to examine clinical scores, their single items, and anamnestic features in their ability to predict 30-day mortality.

**Materials and Methods:**

A retrospective, single-center study from 06/2005 to 01/2010 was performed. Inclusion criteria were presence of pulmonary embolism, availability of patient records and 30-day follow-up. The following clinical scores were calculated: Acute Physiology and Chronic Health Evaluation II, original and simplified pulmonary embolism severity index, Glasgow Coma Scale, and euroSCORE II.

**Results:**

In the study group of 365 patients 39 patients (10.7%) died within 30 days due to pulmonary embolism. From all examined scores and parameters the best predictor of 30-day mortality were the Glasgow Coma scale (≤ 10) and parameters of the circulatory system including presence of mechanical ventilation, arterial pH (< 7.335), and systolic blood pressure (< 99 mm Hg).

**Conclusions:**

Easy to ascertain circulatory parameters have the same or higher prognostic value than the clinical scores that were applied in this study. From all clinical scores studied the Glasgow Coma Scale was the most time- and cost-efficient one.

## Introduction

Acute pulmonary embolism (PE) is a common disease. It has a high mortality in some patient groups, e.g. in patients with hemodynamic instability or right ventricular dysfunction [1].

It is undisputed that identification of high-risk patients is vital. However, the tools of risk stratification are under continued research and improvement. The tools include clinical scores [2] and anamnestic features [3].

Imaging techniques [4] and biomarkers [5] also play a major role in risk stratification. However, these were not considered in the present study.

Clinical scores can be time consuming to ascertain. For example, the euroSCORE is calculated from about 24 items [6]. Furthemore, it can be impossible to gather anamnestic features in patients with a deteriorated state of consciousness.

Therefore, the aim of the present study was to examine a broad array of promising scores, their single items and anamnestic features to find the most time- and cost-efficient risk stratification tools in acute pulmonary embolism.

## Materials and Methods

### Study design

The study was approved by the institutional ethic commitee (Martin-Luther-University). All analyzed patient records were anonymized and de-identified prior to analysis. In a single-center, retrospective study at a hospital of maximum medical care (Halle University Hospital), all patients with acute symptomatic PE were reviewed from 06/2005 to 01/2010.

Patients were identified via analysis of patient records and using the radiological information system. Inclusion criteria were: performance of computed tomography pulmonary angiography positive for pulmonary embolism, availability of patient records and 30-day follow-up. The final study group included 365 patients (age median 65 years, age range 18–91, 178 male). Treatment methodology was same during the study period following the guidelines of the diagnosis and management of acute pulmonary embolism as defined by the European Society of Cardiology [7]. High risk patients underwent thrombolysis or embolectomy.

Survival was defined as surviving the following 30 days after the PE diagnosis. Patients, who died from acute respiratory failure, cardiopulmonary arrest or shock in the absence of other cardiopulmonary diseases were defined as non-survivors. Patients who died from other causes were excluded from the study group: one patient with intracranial hemorrhage, one patient with acute renal failure, and four patients with septic shock.

### Computed tomography examination

The computed tomography scanners used during the study period were two 64-multidetector systems (Somatom Sensation 64, Siemens, Erlangen, Germany; Aquillon 64, Toshiba, Neuss, Germany). In the pulmonary computed tomography angiography examination 60 ml of an iodinated intravenous contrast medium (Solutrast 370 with 370 mg iodine / ml, Bracco Imaging Germany GmbH, Konstanz, Germany) was given at a rate of 2.0 ml / second. Typical imaging parameters were 120 kVp, 150–300 mAs, slice thickness 2 mm, and a pitch of 0.6–1.2 depending on body size. Automatic bolus timing was used with effective delays of 12–25 seconds.

### Clinical scores

A clinical score that has been established for PE survival is the pulmonary embolism severity index (PESI) [2]. It estimates the risk of 30-day mortality in patients with acute PE. It will be referred to as ‘original PESI’ in this work.

The original PESI was simplified in 2010 [8]. In the present work this score will be referred to as ‘simplified PESI’. Both scores were included in the present study.

The Acute Physiology and Chronic Health Evaluation II (APACHE II) is a very common severity-of-disease classification system [9]. It is calculated at admission to an intensive care unit and predicts risk of death. We decided to include the APACHE II because it is widely distributed.

Part of the APACHE II, yet a score of its own, is the revised Glasgow Coma Scale (GCS) [10]. As the APACHE II, it is a widely used score with a 15 point neurological scale that aims to record the conscious state of a patient.

A score that is promising and contains a lot of interesting single items is the euroSCORE II. It has been validated to assess risk factors for mortality in adult patients undergoing cardiac surgery [6]. Its latest definition can be found online (www.euroscore.org).

In addition, all single items of the abovementioned scores were tested on their own in their ability to predict 30-day mortality.

Single items of the original and simplified PESI, the euroSCORE II, and the APACHE II were taken from the patient records. The scores were calculated according to the description in the original reports [2,6,8–10].

Some items were contained in more than one score (e.g. age, gender, NYHA, heart rate, respiratory rate, blood pressure, history of cancer).

The three single items of the euroSCORE II that were not applicable to patients with PE were urgency (of surgery), risk of the intervention (of surgery), and surgery on thoracic aorta (yes / no). For calculation purposes, all three items were defined to be 0 (i.e. to have no influence on the score) in all study patients.

### Anamnestic features

Anamnestic features that were promising to us are: presence of hemoptysis, acute onset of dyspnea, presence of chronic obstructive pulmonary disease, and presence of any form of anticoagulation prior to PE.

Furthermore, we examined whether admission at night or at weekend influenced prognosis. The information on the day and time of admission was taken from the time at which the patient’s insurance card was read. When the card was read between 8:00 a.m. and 4:00 p.m. the patient was considered as ‘admitted at day shift’. All anamnestic features were taken from patient records.

### Statistical analysis

Collected data were evaluated by means of descriptive statistics: absolute and relative frequencies, median and 25%–75% interquartile range, sensitivity, specificity, negative predictive value, and positive predictive value.

The two-tailed Mann-Whitney-U test was used to estimate statistical differences between groups for metric variables. The chi-square-test was used to estimate statistical differences between groups for binary variables (SPSS Version 18, IBM, New York).

The suggested cut off points for measurements to predict 30-day mortality were selected so that the Youden index (sensitivity + specificity − 1) had a maximum value.

## Results

In the study group of 365 patients 39 patients (10.7%) died within 30 days due to PE (14 patients respiratory failure due to PE; 25 patients cardiopulmonary arrest due to PE).

### Clinical scores

The clinical scores were calculated for each patients. The median of the results for the group of survivors and non-survivors is shown in Table 1. The APACHE II, the GCS, and the original PESI were significantly different in survivors and non-survivors.

**Table 1:** Clinical scores in survivors and non-survivors (30-day mortality in patients with acute pulmonary embolism). Scores are sorted in order of ascending p-value.

	survivor* n = 326	non-survivor* n = 39	p-value
APACHE II	10 (6–15)	25 (14–32)	<0.0001
Glasgow Coma Scale	15 (15–15)	8 (3–15)	<0.0001
original PESI	109 (79–140)	168 (138–203)	<0.0001
simplified PESI	2 (1–3)	2 (2–3)	0.0011
euroSCORE II	2.0 (1.0–6.7)	6.5 (2.7–12.5)	0.0026

*Median (25%–75% interquartile range)

**Fig 1:**
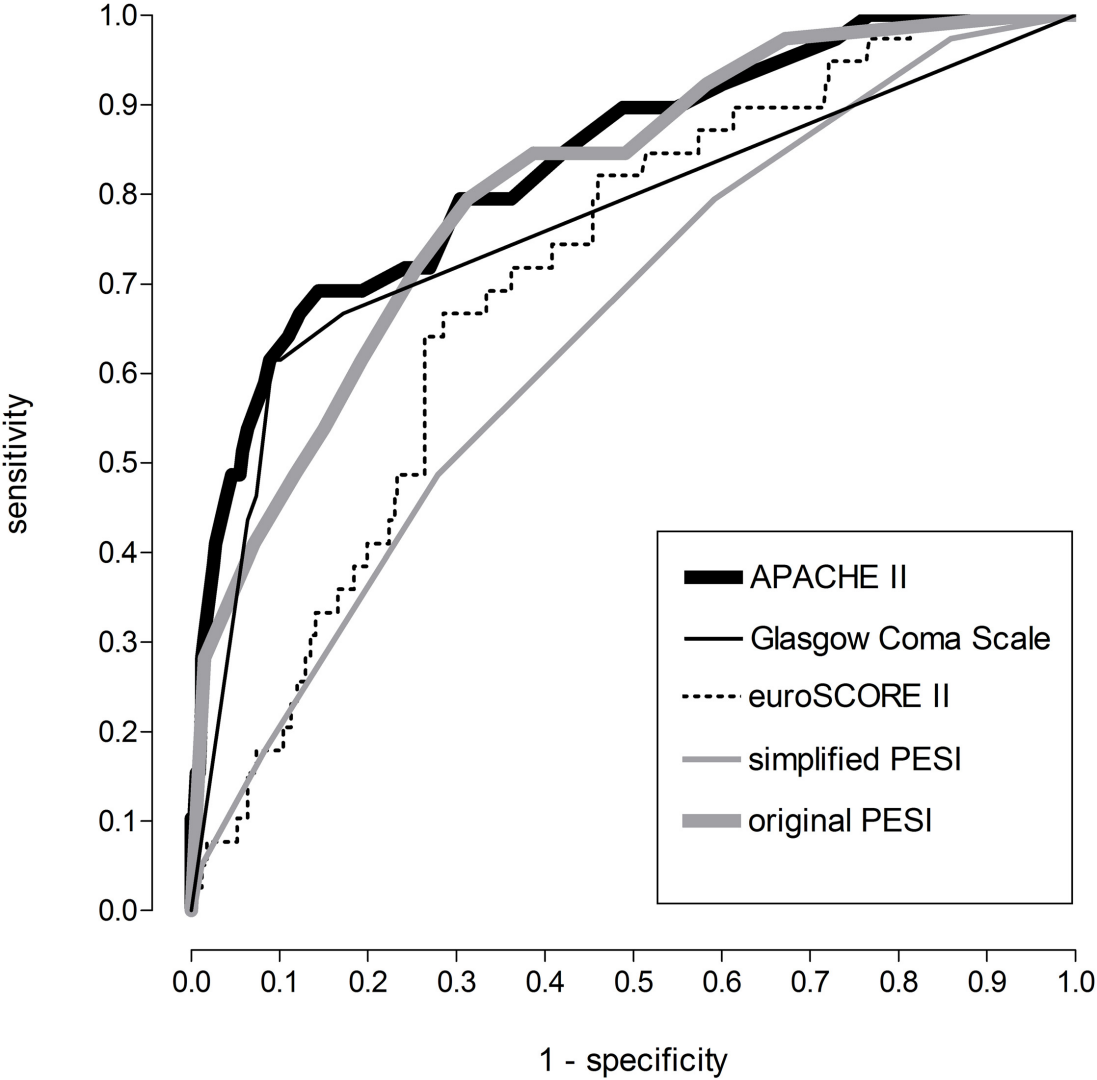
Receiver operating characteristic curve of clinical scores and their ability to predict 30-day mortality on patients with acute pulmonary embolism.

To compare the clinical scores in their ability to predict 30-day mortality a receiver operating characteristic curve was computed (Fig 1). The optimal cut off to predict 30-day mortality according to the Youden index, as well as sensitivity, specificity, positive and negative predictive value were calculated for each score. The results are shown in Table 2. The APACHE II performed best, the simplified PESI performed worst regarding their prognostic value on 30-day mortality in patients with PE.

**Table 2:** Prediction of 30-day mortality by clinical scores. Scores are sorted in order of descending Youden index.

	cut off	sensitivity	specificity	PPV	NPV	Youden index
APACHE II	≥ 19	69%	86%	37%	96%	55%
Glasgow Coma scale	≤ 10	62%	91%	44%	95%	52%
original PESI	≥ 150	82%	67%	23%	97%	50%
euroSCORE II	≥ 5.9	64%	74%	23%	94%	38%
simplified PESI	≥ 3	49%	72%	17%	92%	21%

Abbreviation: PPV positive predictive value; NPV negative predictive value

In addition, all single items of the scores were computed for each patient. The median of the results for the group of survivors and non-survivors is shown in Table 3. Eight single items (blood pressure, respiratory rate, pH arterial, post cardiac massage, mechanical ventilation, inotropes, acute renal failure) were significantly (p < 0.0001) different between survivors and non-survivors.

**Table 3:** Single items of scores in survivors and non-survivors (30-day mortality in patients with acute pulmonary embolism). Items are sorted in order of ascending p-value.

	survivor* (n = 326)	non-survivor* (n = 39)	p-value
mean arterial pressure [mm Hg]	80 (73–91)	66 (56–79)	<0.0001
systolic blood pressure [mm Hg]	111 (100–125)	94 (78–111)	<0.0001
respiratory rate [breaths per minute]	23 (21–23)	25 (23–31)	<0.0001
pH arterial []	7.431 (7.400–7.475)	7.334 (7.156–7.468)	<0.0001
critical preoperative state [yes / no] †			
» post cardiac massage [yes / no]	6% (19)	56% (22)	<0.0001
» mechanical ventilation [yes / no]	12% (38)	77% (30)	<0.0001
» inotropes or intra aortal balloon pump [yes / no]	13% (42)	67% (26)	<0.0001
» acute renal failure (anuria or oliguria <10ml / hour) [yes / no]	6% (19)	28% (11)	<0.0001
» ventricular tachycardia or ventricular fibrillation or aborted sudden death [yes / no]	13% (44)	28% (11)	0.0152
heart rate [beats per minute]	106 (90–119)	120 (100–130)	0.0018
potassium K+ (serum) [mmol/l]	4.1 (3.8–4.6)	4.3 (4.0–5.1)	0.0034
Chronic health points †			
» organ insufficiency [yes / no]	54% (175)	77% (30)	0.0057
» post emergency surgery [yes / no]	8% (25)	18% (7)	0.0319
» no previous surgery [yes / no]	77% (252)	69% (27)	0.2617
» post elective surgery [yes / no]	17% (57)	18% (7)	0.9425
left ventricular ejection fraction (LVEF) †			
» LVEF ≥ 50%	69% (226)	51% (20)	0.0231
» LVEF < 50%	31% (100)	49% (19)	0.0231
» LVEF < 30%	6% (21)	15% (6)	0.0437
» LVEF < 20%	2% (7)	5% (2)	0.2566
chronic lung disease [yes / no]	17% (54)	31% (12)	0.0293
creatinine [μmol/l]	92 (76–111)	120 (86–154)	0.0915
Saturation [%]	92 (87–97)	92 (85–98)	0.0440
extracardiac arteriopathy [yes / no] †			
» claudication [yes / no]	6% (20)	13% (5)	0.1182
» previous or planned intervention on the abdominal aorta, limb arteries or carotids [yes / no]	10% (31)	13% (5)	0.5121
» carotid occlusion or >50% stenosis [yes / no]	3% (10)	3% (1)	0.8620
» amputation for arterial disease [yes / no]	0% (0)	0% (0)	N/A
angina at rest [yes / no]	14% (46)	5% (2)	0.1167
hematocrit [%]	38 (32–41)	35 (31–40)	0.1491
age [years]	63 (53–75)	67 (58–76)	0.1533
poor mobility [yes / no]	17% (55)	26% (10)	0.1760
active endocarditis [yes / no]	1% (2)	3% (1)	0.2022
History of cancer [yes / no]	29% (96)	38% (15)	0.2475
white blood cell count (x1000)	10.0 (8.5–12.5)	13.5 (10.0–18.6)	0.3016
NYHA †			
» NYHA I	16% (51)	10% (4)	0.3740
» NYHA II to IV	43% (140)	44% (17)	0.9387
» NYHA III to IV	27% (88)	21% (8)	0.3849
» NYHA IV	11% (35)	13% (5)	0.6937
sodium Na+ (serum) [mmol/l]	138 (136–140)	138 (134–140)	0.4056
temperature (rectal) [°C]	37.0 (36.9–37.5)	37.0 (36.5–37.8)	0.5034
diabetes [yes / no]	29% (93)	33% (13)	0.5320
previous cardiac surgery [yes / no]	8% (26)	10% (4)	0.6240
recent myocardial infarction [yes / no]	6% (21)	8% (3)	0.7658
severe pulmonary hypertension (> 55 mm Hg) [yes / no]	14% (47)	13% (5)	0.7874
Male sex [yes / no]	49% (159)	49% (19)	0.9948

*Median (25%–75% interquartile range) of metric variables and percent (number) of categorical variables.

^†^ subitems (grey underground) were kept together and ranked according to the subitem with the lowest p-value.

For single items of the scores that showed a highly significant difference between groups (p < 0.0001) an optimal cut off to predict 30-day mortality according to the Youden index was calculated. The results are shown in Table 4.

**Table 4:** Prediction of 30-day mortality by single items of clinical scores. Items are sorted in order of descending Youden index.

	cut off	sensitivity	specificity	PPV	NPV	Youden index
» mechanical ventilation [yes / no]	yes	77%	88%	44%	97%	65%
» inotropes or intra aortal balloon pump [yes / no]	yes	67%	87%	38%	96%	54%
» post cardiac massage [yes / no]	yes	56%	94%	54%	95%	51%
pH arterial []	< 7.335	54%	92%	44%	94%	46%
mean arterial pressure [mm Hg]	< 67	56%	84%	30%	94%	41%
systolic blood pressure [mm Hg]	< 99	56%	78%	24%	94%	35%
respiratory rate [breaths per minute]	≥ 25	51%	83%	26%	93%	34%
acute renal failure (anuria or oliguria <10ml / hour) [yes / no]	yes	28%	94%	37%	92%	22%

Abbreviation: PPV positive predictive value; NPV negative predictive value

### Anamnestic features

Anamnestic features were computed for each patient. The median of the results for the group of survivors and non-survivors is shown in Table 5. Anamnestic feautures were not significantly different between survivors and non-survivors. Therefore, no cut off values were calculated.

**Table 5:** Anamnestic features in survivors and non-survivors (30-day mortality in patients with acute pulmonary embolism). Features are sorted in order of ascending p-value.

	survivor* (n = 326)	non-survivor* (n = 39)	p-value
acute Dyspnea [yes / no]	53% (173)	69% (27)	0.0552
chronic obstructive pulmonary disease [yes / no]	11% (36)	18% (7)	0.2061
Admitted at weekday [yes / no]	79% (257)	85% (33)	0.3984
hemoptysis [yes / no]	6% (18)	3% (1)	0.4320
Admitted at day shift [yes / no]	57% (186)	54% (21)	0.7022
Oral anticoagulation prior to PE [yes / no]	36% (118)	36% (14)	0.9707

* percent (number)

## Discussion

A recent meta-analysis [3] found an overall mortality of 6.5% in 2.288 patients. In some studies mortality rates of up to 18% [11] were described. Another meta-analysis with about 20.000 patients described a mortality of 10.7% [12]. Therefore, the mortality found in the present study confirms findings of the literature.

### Clinical scores

The present results confirm the prognostic value of the original PESI. The optimal cut off found in the present study marks patients of the highest risk class in the original definition of the score [2]. Contrary to other studies [8], the simplified PESI did not performed equally well compared to the original PESI. Again, the optimal cut off found in the present study marks patients of high risk according to the original definition of the score [8].

The scores that were specifically developed to predict 30-day mortality in PE did not perform superior in our study group. We attribute this to the lack of specificity (see also Table 2). The PESI was derived from a patient population of 186 hospitals [2]. In contrast, the present results were derived from a hospital of maximum medical care which results in a higher fraction of patients with severe co-morbidities. However, co-morbidities result in a higher risk class in the PESI, making the score less specific in the present patient group.

The euroSCORE II that is validated in assessing mortality risk in cardiac surgical adult patients [6] had an inferior prognostic value in patients with PE compared to the original PESI. In addition, the score is computed from a large number of single items. Therefore, we do not suggest using it in clinical routine for determining the prognosis in patients with PE. In retrospect, the score performed worse than expected in the present study. Nevertheless, the calculation of the score was useful because a number of single items of the score turned out as very good prognostic parameters.

The APACHE II predicts risk of death and is calculated at admission to an intensive care unit. It is remarkable, that the score performed slightly better than the original PESI in determining mortality in patients with PE. The optimal cut off found in the present study marks a mortality of 25% and higher according to the original definition of the score [9].

We assume that the good performance of the APACHE II is based on two facts. Firstly, the score is not devoted to a specific disease (e.g. contrary to the euroSCORE II that is devoted to cardiac surgical adult patients). Therefore, it can track risk factors that are independent of specific disease features. Secondly, the APACHE II was derived from intensive care unit patients. These patients usually have a high number of severe co-morbidities. Therefore, the patient group in the present study might have been similar to the patient group from which the APACHE II was derived.

The Glasgow Coma Scale (GCS) performed equally well in predicting mortality compared to the APACHE II and the original PESI. The scale has been established for predicting mortality after traumatic brain injury and it is an item in a considerable number of other scores devoted to prognosis [9,13]. However, no studies on the use of the Glasgow Coma Scale alone in predicting 30-day mortality in PE have been done before as far as we know.

Some single items of scores performed equally well or even better in predicting mortality compared to the best clinical scores. Contrary to clinical scores, single items are much faster to ascertain. The best three of them (mechanical ventilation, post cardiac massage, inotropes or intra aortal balloon pump) have a high specificity. However, they cannot be applied to less severely ill patients.

In this case, the other variables may be helpful: arterial pH, respiratory rate and blood pressure are fast, cheap and reliable to measure. The GCS and the abovementioned single items have in common that they are indicators of a threatening circulatory or conscious state. In addition, they are independent from specific disease feature. The advantage of these simple measurements is maybe, that they are “too dumb to fool”.

The high prognostic value of simple measurements has also previously been reported [14].

### Anamnestic features

It is known that PE presents more significantly in patients with chronic obstructive pulmonary disease than in others [15]. However, in our study group it had no significant influence on prognosis.

The presence of acute dyspnea compared to slowly progressing dyspnea could be an indicator of more severe PE. The present data supported this thesis. Yet, differences were not significant.

It is to presume that on the one hand, the use of any form of anticoagulation prior to PE indicates a pre-existing morbidity resulting in a higher mortality. On the other hand, the anticoagulation should work against more severe PE. None of these speculations was proven by the present data.

It is a sign of quality that patients admitted at night at weekend had statistically a similar prognosis than patients admitted at day at the middle of the week.

## Limitations

Due to the retrospective nature of our study comorbidities were defined solely by provider documentation in the patient chart review. Therefore, undescribed comorbidities could be missed. By excluding patient deaths that were not directly related to PE, complications of PE therapy and PE mortality might be underestimated.

## Conclusion

In a patient group with severe co-morbidities the simplified PESI might be not specific enough. The results of the present study provide support for enhancing efforts toward identifying PE patients at risk of death. The original PESI, the APACHE II, and the Glasgow Coma Scale perform almost equally well in predicting mortality in patients with PE. However, the Glasgow Coma Scale is by far the score that is easiest to ascertain. Nevertheless, a number of circulatory parameters show equally or even better prognostic value. When implementing preventive strategies among patients with PE and severe co-morbidities the results of the present study support the selection of appropriate parameters.
